# ADAR1: “Editor-in-Chief” of Cytoplasmic Innate Immunity

**DOI:** 10.3389/fimmu.2019.01763

**Published:** 2019-07-25

**Authors:** Mart M. Lamers, Bernadette G. van den Hoogen, Bart L. Haagmans

**Affiliations:** Department of Viroscience, Erasmus MC, Rotterdam, Netherlands

**Keywords:** ADAR1, cytoplasmic innate immunity, PKR, MDA5, OAS, RIG-I

## Abstract

Specialized receptors that recognize molecular patterns such as double stranded RNA duplexes—indicative of viral replication—are potent triggers of the innate immune system. Although their activation is beneficial during viral infection, RNA transcribed from endogenous mobile genetic elements may also act as ligands potentially causing autoimmunity. Recent advances indicate that the adenosine deaminase ADAR1 through RNA editing is involved in dampening the canonical antiviral RIG-I-like receptor-, PKR-, and OAS-RNAse L pathways to prevent autoimmunity. However, this inhibitory effect must be overcome during viral infections. In this review we discuss ADAR1's critical role in balancing immune activation and self-tolerance.

## Introduction

Our innate immune system has evolved to specifically recognize common molecular patterns formed as a result of virus replication. These molecular patterns—commonly referred to as pathogen-associated molecular patterns or PAMPs—are detected by specialized receptors, called pattern recognition receptors (PRRs), which can rapidly trigger the immune system after ligand recognition. The replication of virtually all RNA viruses and even dsDNA viruses generates long, perfectly base-pairing double stranded (ds) RNA intermediates in the cytoplasm. As these dsRNAs are unusual in the cytoplasm of eukaryotic cells, sensory systems have evolved to detect this PAMP. These systems then signal the presence of an invading virus, allowing the cell to take appropriate measures. The main system detecting cytoplasmic dsRNA is the RIG-I-like receptor (RLR) signaling pathway, named after the first cytoplasmic PRR discovered: retinoic acid-inducible gene I (1, 2) (RIG-I). Subsequently, another related protein, melanoma differentiation-associated gene 5 (MDA5), was also found to be involved in dsRNA sensing (3). RLR signaling results in the production of the antiviral type I interferons (IFN) after PRRs oligomerize along dsRNA filaments. These oligimerized PRRs recruit the scaffolding protein mitochondrial activation signaling (MAVS) and IKK-related kinases and this ultimately results in the phosphorylation of the transcription factors interferon regulatory factor 3 and 7 (IRF3 and IRF7) (4, 5). These transcription factors then move to the nucleus where they activate type I IFN promoters leading to the secretion of IFNs (6). Next, IFN receptor signaling leads to the induction of an antiviral state in the producing and neighboring cells through the upregulation of interferon stimulated genes (ISGs), which encode proteins with direct antiviral effector functions, such as protein kinase R (PKR), 2′-5′-oligoadenylate synthetase 1 (OAS1), and RNAse L.

While PRR activation and IFN production are tightly controlled in order to prevent false triggering of the immune system, it is becoming clear that certain endogenous RNAs can also form dsRNAs. This includes RNAs transcribed from retrotransposons, such as Alu repeats, and RNAs originating from the mitochondrial matrix (7). Although these RNAs have the correct structure to activate PRRs, they appear not to do so under normal circumstances as this would lead to severe autoimmune disease (8). This raises the question as to how cells distinguish between self and non-self nucleic acids.

One process that may be involved in the discrimination between self and non-self nucleic acids is the editing of RNAs. RNA editing is a process that regulates and expands the diverse functions of RNA transcripts and several types of editing have been characterized so far. In mammals, two forms of RNA editing exist, the deamination of cytosines to uracils by members of the apolipoprotein B mRNA editing enzyme catalytic subunit (APOBEC) protein family, and the deamination of adenosines to inosines (A-to-I editing) by the adenosine deaminase acting on RNA (ADAR) gene family. The cell's translation and splicing machineries interpret inosines as guanosines, instead of the adenosines encoded in the genome (9). This can lead to non-synonymous substitutions if editing takes place in coding sequences (10). More importantly, A-to-I substitutions have the capacity to destabilize dsRNA structures formed between complementary strands due to the replacement of Watson–Crick AU base pairs by IU wobble pairs, which are isomorphic with GU base pairs (11).

Mounting evidence suggests that cytoplasmic antiviral immunity is controlled at the level of dsRNA recognition by RNA editing, which appears to play a pivotal role in maintaining self-tolerance and preventing autoimmunity. Intriguingly, mutations in the adenosine deaminase ADAR1 can confer autoimmunity in humans and in mice models (12–17) and recently ADAR1 has been shown to regulate the canonical RLR-, PKR-, and OAS-RNAse L pathways. This review summarizes recent advances related to the function of ADAR1 as a “master regulator” of cytoplasmic innate immunity and discusses how the host can still mount an effective antiviral response in the presence of ADAR1.

## ADAR1: RNA Editor Induced by IFN

**Figure 1:**
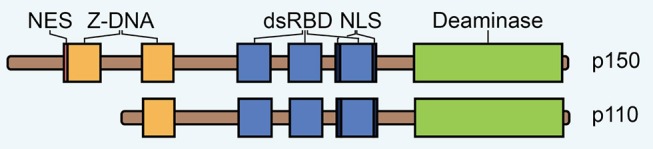
The domain architecture of ADAR1 isoforms p150 and p110. NES, nuclear export signal; dsRBD, double-stranded RNA binding domain; NLS, nuclear localization signal.

A-to-I editing was originally discovered as enzymatic activity unwinding dsRNA in *Xenopus laevis* (18, 19). The protein responsible for this activity, now known as ADAR1, was termed an RNA unwindase, and shortly thereafter it was found to also possess A-to-I editing activity (20–24). In addition, two other ADARs were discovered, thus comprising a gene family of three members in mammalian genomes (10, 25–28). Two encode for ubiquitously expressed enzymes with adenosine deaminase activity, the IFN-inducible ADAR1 and the constitutively expressed ADAR2 (24, 29, 30). The third, ADAR3, has not been shown to possess any enzymatic activity and is expressed primarily in the brain (30–32). ADAR proteins show extensive architectural similarity, with centrally located three repeated copies of a dsRNA-binding domain (dsRBD) and a single deaminase domain at the C-terminus (Figure 1). The presence of these dsRBDs underlines that ADAR activity is directed toward dsRNA.

ADAR1 is expressed to higher levels than the other ADARs and it is responsible for the majority of editing activity (30). The protein encoded by this gene exists in two forms that are generated from alternative exon 1 structures that initiate from different promoters (33). A small isoform (p110) is expressed constitutively while a larger isoform (p150) is upregulated in response to IFN. The p110 isoform is a truncated version of p150, lacking one Z-DNA binding domain at the N-terminus, which contains a nuclear export signal (NES) (33, 34) (Figure 1). Therefore, p110 is almost exclusively found in the nucleus while p150 is largely expressed in the cytoplasm (35–38). A bimodal nuclear localization signal (NLS) flanking the third dsRBD mediates nuclear import via transportin-1 (39).

## ADAR1 Blocks RIG-I-Like Receptor Signaling

The promoter responsible for p150 expression possesses a consensus interferon-stimulated response element (ISRE) characteristic of ISGs (33, 35, 40). The discovery of this element promised an important role for ADAR1 in the immune response soon after the protein was discovered. The importance of ADAR1 for survival was already recognized early on as *Adar1*^−/−^ mice die by embryonic day 11.5–14 with widespread apoptosis and cell death of liver hemotopoietic cells (41–43). Clues to the mechanism underlying this lethality, however, could only be understood after the discovery of RLR signaling. In 2009, Hartner and colleagues found that the mortality in *Adar*^−/−^ embryonic mouse model was associated with the overexpression of IFN (44), and three studies in 2014–2015 showed that lethality can be rescued to live birth by deleting either *Mavs* or *Mda5* as well (13, 15, 16). In contrast, *Rig-I* deletion did not rescue lethality and inflammatory responses of *Adar*^−/−^ embryos (16). These findings indicated that in mice ADAR1 is able to block signaling through the MDA5-MAVS axis of the type I IFN signaling pathway, and that MDA5 is stimulated by endogenous dsRNA in ADAR1's absence. Knock-in of an editing-deficient form of ADAR1 did not rescue lethality, indicating that the enzymatic activity is crucial for survival (15). While *Rig-I* deletion in mice did not rescue embryonic lethality, several *in vitro* studies in mice and human cells have suggested that RIG-I activation is also blocked by ADAR1 (45–47). Notably, Yang et al. (45) described that the inhibitory effect of ADAR1 on RIG-I was mediated through RNA binding rather than editing activity. Differences in cell types, including HEK-293T cells, murine embryonic cells, macrophages, and hepatocytes may explain this discrepancy, as it is likely that the involvement of ADAR1 in RLR signaling is cell type dependent.

The discussed findings indicate that the dampening of RLR signaling is crucial to normal homeostasis and that endogenous RNA species can trigger RLRs in the absence of ADAR1. In agreement with the embryonic *Adar*^−/−^ mouse model, naturally occurring mutations in ADAR1 were found in humans with a severe and rare type I interferonopathy, termed Aicardi Goutieres syndrome (AGS) (12, 14). AGS is a fatal childhood encephalopathy characterized by uncontrolled IFN expression and IFN presence in cerebral spinal fluid, giving rise to symptoms reminiscent of a viral infection (48–51). Mutations in MDA5 have also been found in AGS patients (52, 53). These mutations were recently shown to enhance MDA5 activity by increasing its efficiency in recognizing dsRNA (54). A key question arose from this work: “What endogenous ligand is recognized by RLRs that triggers their activation in the absence of ADAR1 and viral replication?”

With the development of deep-sequencing this question could be addressed, for example by screening for editing sites globally. Such efforts showed that mobile genetic elements termed Alu repetitive elements, which can form dsRNA structures when transcribed as inverted Alu repeats, are the most frequent targets of A-to-I editing. Notably, Alu repeats are numerous: there are more than 1 million of these repeats present in the human genome, accounting for almost 11% of its size (55). Alu repeats are likely to be selectively edited due to their secondary structure and size, as ADAR1 editing does not require a strict consensus sequence [reviewed by: (56)]. A-to-I editing in Alu repeats disappears in *ADAR1* knockout (KO) cells, possibly providing an unedited, immunostimulatory dsRNA substrate for RLR signaling (54, 57–71). Most of these Alu repeats are present in introns and 3′ untranslated regions (UTRs) of mRNAs and are transcribed by polymerase-II (54, 71). Mice do not possess the primate-specific Alu repeats, but it is conceivable that other mobile genetic elements capable of forming dsRNA, such as the closely related rodent B1-SINE, could stimulate MDA5 in similar ways if unedited. Additional substrates for ADAR1 editing may be mitochondrial dsRNAs (mtdsRNAs), as it was recently shown that patients with biallelic hypomorphic mutations in the *PNPT1* gene, which encodes polynucleotide phosphorylase PNPase, contain mtdsRNAs in the cytoplasm that can activate MDA5 and induce IFN (7). Deep-sequencing of PNPase depleted cells revealed that mitochondrial RNAs were A-to-I edited. Concurrent depletion of PNPase and ADAR1 enhanced IFN expression, suggesting that ADAR1 editing of these endogenous RNAs blocks IFN activation. Editing of host dsRNAs could differentiate them from viral RNAs by altering their secondary structure to abrogate the formation of extended dsRNA duplexes, preventing IFN activation in uninfected cells (72). Alternatively, edited dsRNA could act as an active inhibitor of IFN signaling. Vitali et al. (73) found that extensively edited dsRNA (IU-dsRNA) inhibits activation of the IFN pathway induced by the dsRNA mimic polyriboinosinic:polyribocytidylic acid (poly(I:C)) *in vitro*. As a mechanism the authors proposed that IU-dsRNA could bind to RLRs with higher affinity than poly(I:C), thereby preventing their activation. Mapping of A-to-I editing sites have shown that editing can be highly concentrated providing a source of the potential IFN-inhibitory IU-dsRNA *in vivo* (15). These data might indicate that ADAR1 is required to provide the cell with a ligand that actively inhibits IFN signaling (and possibly other cytoplasmic antiviral responses), a hypothesis that deserves continued investigation.

## ADAR1 Blocks PKR-Induced Translation Arrest

A protein that is similar to ADAR1 in terms of its induction by IFN and presence of multiple dsRBDs, is the IFN inducible double-stranded RNA-activated protein kinase (PKR). PKR is one of the best-studied ISGs and its expression inhibits the replication of a wide range of viruses (74). It is also antagonized by several viruses, including influenza A virus, vaccinia virus and Ebola virus. It is a member of the eIF2α family of protein kinases that also includes the PKR-like endoplasmic reticulum kinase (PERK), the general control non-depressible 2 kinase (GCN2) and the hemin-regulated inhibitor of translation (HRI). Each of these kinases is activated under different conditions of cellular stress, which is cytoplasmic dsRNA in the case of PKR. Substrate recognition leads dimerization via its dsRBDs (75, 76), autophosphorylation and subsequent phosphorylation of eIF2α (77, 78), which shuts down 5′-cap-dependent mRNA translation to prevent viral protein synthesis (79, 80). This is accompanied by the formation of cytoplasmic stress granules (SGs), dense aggregations of RNA and proteins that store stalled translation pre-initiation complexes.

Besides its function in dampening IFN induction, ADAR1 is also known to block translation arrest and stress granule formation by inhibiting PKR activation (81–86). Several studies have shown that ADAR1 functions in a proviral manner via RNA editing and inhibiting PKR. Examples include measles virus (MV) (83), vesicular stomatitis virus (VSV) (81, 85), and human immunodeficiency virus (HIV) (82, 87, 88). In these cases, ADAR1 is able to block PKR and this inhibition was found to occur through both editing-dependent and independent mechanisms. For MV, ADAR1-deficiency was shown to lead to elevated cytotoxicity and apoptosis upon virus infection, while viral replication was decreased (83). Moreover, PKR activation observed in ADAR1 knockdown (KD) cells upon MV infection was not rescued by an editing deficient form of p150, indicating that for MV PKR inhibition is editing-dependent (89). In contrast, during VSV and HIV infection, ADAR1's inhibitory effect on PKR was found to occur in an editing-independent fashion (81, 82). In the case of HIV, an increased interaction between ADAR1 and PKR was observed during infection, which could represent competition between both proteins for the same substrate, or could indicate that ADAR1 directly interacts with PKR to prevent its dimerization and subsequent autophosphorylation. Complex formation between ADAR1 and PKR is independent of RNA and the first dsRBD of ADAR1 is required for the binding (81). By interfering with the activation of PKR, ADAR1 prevents the phosphorylation of eIF2α and the formation of SGs, and consequently allows the translation of viral mRNAs (81, 83–85).

The observation that *Mavs* and *Adar1* double knockout (DKO) mice still die shortly after birth suggests that the lethality of *Adar1*^−/−^ mice is not solely reliant on IFN induction. Suppressing PKR may also be required for survival, even in the absence of viral infection. Interestingly, a recent study sheds more light on this (71). In this study, ADAR1 KO (either ADAR1 or ADAR1 p150) was shown to decrease protein, but not mRNA, levels of several ISGs following IFN treatment. This was associated with PKR and eIF2α phosphorylation upon IFN treatment in KO, but not in wild-type (WT) cells. Furthermore, PKR KD in ADAR1 KO cells partially restored ISG protein levels, indicating that PKR activation in ADAR1 KO cells is responsible for the lack of ISG protein expression. Intriguingly, in IFN-treated ADAR1 KO cells, phosphorylated PKR levels were significantly reduced by transcription inhibition, suggesting that an endogenous RNA species, transcribed in response to IFN, is responsible for the activation of PKR in these cells. In this same study, it was found that >90% of A-to-I editing takes place in Alu repeats, suggesting that ADAR1 editing or binding inhibits them from activating PKR. Altogether, these results indicate that ADAR1 is required for maintaining efficient translation during the IFN response and that endogenous RNA transcripts activate PKR in ADAR1's absence. The endogenous RNA species that activate PKR could be the same RNAs that activate MDA5 in the absence of ADAR1, but this remains to be tested.

The relevance of the findings by Chung and colleagues was further underlined by generating ADAR1 KO human embryonic stem cells (hESC). While this genetic permutation was not lethal to these hESCs, they did exhibit spontaneous MDA5-mediated IFNβ production, PKR activation, and apoptosis upon differentiation to neural progenitor cells (NPCs), indicating that requirements for ADAR1-mediated PKR or MDA5 inhibition depend on cell type or differentiation state. Also, the fact that NPCs are more vulnerable to the effects of ADAR1 deficiency may indicate why AGS is associated with neurological abnormalities. These results are in agreement with a study by Yang and colleagues, in which ADAR1 KO was induced in newborn mice. Upon sacrifice, these inducible ADAR1 KO mice showed high levels of IFN specifically in neuronal tissues (45). Unfortunately, neither studies showed whether the observed effects were PKR- or MDA5-mediated.

In agreement with the finding that SGs can function as platforms for RLR signaling (90), mounting evidence suggests that PKR is also involved in the induction of type I IFN (91). PKR may be involved in the activation of NFκB, a transcription factor required for efficient IFN induction, possibly through its interaction with the IKK complex (92). Moreover, PKR was shown to bind members of the TRAF family, which are involved in MAVS signaling (93). Further, several studies have shown that PKR is required for IFN production in response to poly(I:C) *in vitro* (94–97). PKR is also required for the production of type I IFN in response to a subset of viruses, including encephalomyocarditis virus, Theiler's murine encephalomyelitis virus, MV, West Nile virus, and Semliki forest virus, but not influenza and Sendai virus (98–102). In addition, a recent study showed that PKR interacts directly with MDA5 and is able to enhance MDA5-mediated IFN production (103). Efficient IFN induction required the catalytic activity of PKR, but not the phosphorylation of eIF2α suggesting that these effects are independent of the induction of SGs or translational shutdown. Although ADAR1 is able to inhibit PKR activation, the contribution of this inhibition to PKR-mediated type I IFN induction remains to be determined.

## ADAR1 Blocks the OAS-RNAse L Pathway

The IFN-inducible oligoadenylate synthetase (OAS)-RNase L pathway is activated upon sensing of dsRNA. OAS proteins (OAS1, OAS2, OAS3) produce 2′,5′-oligoadenylates (2-5A) upon dsRNA recognition. This second messenger activates RNase L by binding with high affinity to the inactive, monomeric form of RNAse L, causing it to dimerize into its enzymatically active state (104). In this state it is able to cleave both viral and host ssRNA, predominantly after UpU and UpA dinucleotides, leaving a 5′OH and a 2′,3′-cyclic phosphate on the cleavage products (105). RNAse L activation can lead to translation arrest through cleavage of ribosomal RNA and mRNA (79, 106), autophagy (107, 108), and apoptosis (109–111), preventing viral replication, and spread.

A recent study reported that the cell-lethal phenotype of ADAR1 deletion in human lung adenocarcinoma A549 cells was rescued by RNASEL KO. This indicated that the OAS/RNase L pathway is the primary mechanism that leads to cell death in these cells, in the absence of ADAR1 and even in the presence of MDA5 and MAVS (112). Furthermore, ectopic expression of active, but not inactive, RNase L, in ADAR1/RNASEL DKO cells promoted cell death, which supports the central role of RNase L activation in dsRNA-mediated cell death in this cell line. In addition, IFN-induced 2-5A accumulation was higher RNASEL-ADAR1 DKO cells than in WT and RNASEL KO cells, indicating that ADAR1 prevents the activation of OAS. Moreover, these results suggest that ADAR1 is the primary regulator of RNAse L activation in this cell type, most likely by preventing OAS activation. Whether these data could be extrapolated to other cell lines or even the entire organism remains to be tested, but RNase L-mediated cell death in the absence of ADAR1 could potentially contribute to AGS. Furthermore, the findings in *Adar* and *Mavs/Mda5* DKO mice and in ADAR1 KO NPCs suggest that not the OAS-RNAse L but the RLR signaling pathway leads to cell death in absence of ADAR1. Although it is uncertain at present which pathways downstream of RLR signaling are mediating lethality, these data indicate that the OAS-RNAse L pathway be an important mediator of cell death.

## ADAR1 and Viruses

A bias toward A-to-G and U-to-C mutations has been described for a wide range of genetically diverse viruses, suggestive of ADAR editing (113). This could potentially lead to the synthesis of dysfunctional viral proteins and RNA structures. Although several studies have reported high editing levels that suggest a mutagenic and antiviral role for ADAR1 (113), evidence of an important role for ADAR1 as part of the innate immune response against virus infections is lacking. Additionally, studies that have observed hypermutation have not yet shown, for example using genetic KOs, that a specific ADAR protein is responsible for editing, nor have they shown that editing itself leads to decreased virus replication. Furthermore, it must be noted that for many viruses it may not be possible to distinguish between the editing of replication-competent viruses and defective interfering (DI) viral genomes, which may be edited frequently (114–116). While editing of viral genomes could decrease infectivity, editing of DI viral genomes may prevent these from activating the innate immune system. Inhibition of ADAR editing has been reported for adenovirus and Vaccinia virus gene products, but it is yet unknown whether RNAs encoded by these DNA viruses are edited by ADARs. The adenovirus-associated (VAI) RNA is responsible for ADAR1 inhibition and interestingly it is also is capable of blocking both PKR and IFN induction (117–119). RNA binding by PKR is generally an activatory signal, but VAI RNA binding to PKR and ADAR1 is thought to sterically inhibit homodimerization and activation (119–121). The vaccinia virus E3L protein like ADAR1 contains a Z-DNA binding domain next to a dsRBD and also blocks IFN induction, PKR, and ADAR1 (95, 121, 122). Notably, viral ADAR1 inhibition could be a by-product of shielding or sequestering dsRNAs from antiviral PRRs, like PKR, OAS, RIG-I, and MDA5.

ADAR1's dampening effects on antiviral systems and its proviral role during the replication of a wide range of viruses (81–83, 85, 87, 88) suggest that there may also be viruses that have evolved to usurp ADAR1 for their replication. In agreement, a recent study showed that the influenza A virus NS1, and dengue virus NS3 can bind ADAR1 and enhance its editing function, although editing of viral genomes was not investigated (123). As these proteins are also capable of blocking IFN induction, this raises the question whether IFN-inhibition is dependent on ADAR1. The modulation of ADAR1 by viruses underlines the importance of this protein in innate immunity and viral replication, although it must be noted that none of these gene products have been demonstrated to alter A-to-I editing through direct agonism or antagonism of ADAR1 during infection.

## ADAR1 and Cancer

Recent advances in the field point toward an important role for ADAR1 in tumorigenesis [reviewed in: (124, 125)]. In most tumor types, RNA editing levels are elevated compared to matched normal tissues, suggesting that editing may supplement genomic DNA alterations and drive tumorigenesis (70). Editing in coding regions of certain mRNAs has been associated with oncogenic activity by giving rise to amino acid changes that alter protein properties (70, 124, 126–129). For example, during esophageal squamous cell carcinoma progression editing of the Antizyme inhibitor 1 mRNA increases, leading to a serine to glycine change that affects cellular polyamine levels. In addition, ADAR1 can also edit microRNAs (miRNAs), which could inhibit their processing or lead to retargeting (130–133). For example, *miR-200b* was found to be overedited in multiple cancer types, leading to retargeting to the tumor suppressor leukemia inhibitory factor receptor. Editing levels of *miR-200b* were found to correlate with poor patient survival. In another study, ADAR1 editing was found to reduce levels of the tumor suppressing *let-7* family of miRNAs, leading to enhanced self-renewal of leukemic stem cells (132). Besides direct effects on miRNA function through editing, ADAR1 has also been shown to interact directly with Dicer to promote processing of siRNAs and miRNAs, RISC loading of miRNAs, and consequently silencing of target RNAs (134). Notably, these effects were independent of ADAR1 editing. These data indicate that loss of ADAR1 could result in dysregulated expression of many genes, which are otherwise silenced by miRNAs. Although ADAR1 is likely to contribute to tumorigenesis by altering gene expression through editing dependent and independent mechanisms, ADAR1 has recently also been shown to contribute to tumorigenesis through its role as regulator of innate immunity.

As reviewed extensively by Parker et al. (135), type I IFNs have important antitumor effects, such as the induction of apoptosis and attraction of infiltrating immune cells. Therefore, malignant cells with dysfunctional IFN responses have a selective advantage. Moreover, the loss of IFN defenses is thought to form the basis for the cancer selectivity of several oncolytic viruses (136). Three recent studies have shown that deleting ADAR1 in tumor cells can induce lethality (137–139). Removal of ADAR1 also rendered these cells more vulnerable to immuno-therapy and overcame resistance to checkpoint blockade, pinpointing ADAR1 as a new immuno-oncology target (138). Interestingly, the lethality of ADAR1 KO tumor cells was rescued by PKR deletion, which is distinct from the embryonic lethality phenotype observed in *Adar1*–/– mice which was mediated through the MDA5/MAVS pathway (13, 15, 16). In another study, lethality of A549 ADAR1 KO cells was mediated through the OAS/RNAse L pathway (112). These data indicate that downstream pathways that mediate cellular lethality after ADAR1 deletion may depend on the cell type, developmental stage, and/or malignant nature of the cells under investigation. However, the loss of ADAR1, either in normal or malignant cells, will lead to the accumulation of unedited endogenous dsRNAs, which trigger cytoplasmic dsRNA sensors. Malignant cells may have elevated levels of unedited dsRNAs due to the loss of suppressive epigenetic modifications in repeat regions, genomic instability or mitochondrial damage due to oxidative stress (140–142), which would select for cells that have higher editing activities. While the activation of cytoplasmic innate immune sensors is normally deleterious, it may be beneficial in the context of cancer treatment by initiating IFN signaling in the tumor microenvironment. Exploitation of this mechanism through intratumoral ADAR1 inhibition or oncolytic virus therapy using viruses that (naturally) encode ADAR1 inhibitors may be promising candidates for cancer treatment.

## ADAR1 Regulation

**Figure 2:**
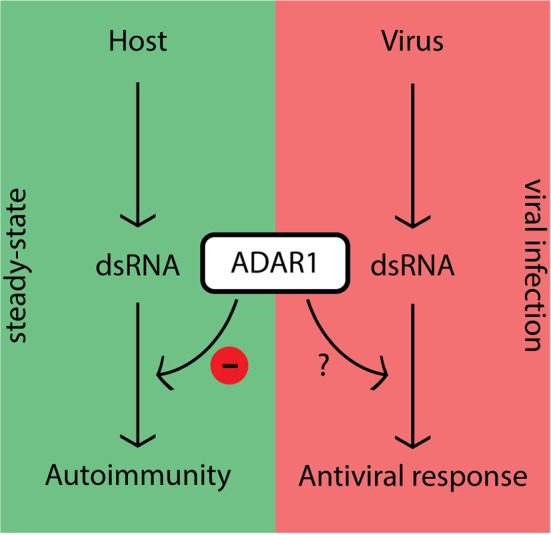
ADAR1's dampening effect on immune activity prevents autoimmunity in steady-state, but should be regulated during viral infection.

The fact that ADAR1 inhibits canonical antiviral pathways in steady-state raises the question: “How does the host overcome this inhibition during viral infection in order to mount an effective antiviral response?” (Figure 2). Although this question is currently under extensive investigation, several lines of evidence suggest that ADAR1 activity is tightly controlled in the cell. The promoter responsible for p150 expression possesses a consensus interferon-stimulated response element (ISRE) characteristic of ISGs (33, 35, 40). In steady-state cells generally express low levels of p110 and p150. These levels may not be capable of editing the large amounts of viral dsRNA intermediates generated during replication, which would leave unedited substrates to be recognized by PRRs once the dsRNA levels exceed a certain threshold. However, as transcription is a relatively slow process other, more rapid, regulatory mechanisms are likely to be in place.

Besides regulation at the transcriptional level by IFN, ADAR1 appears to act as a dimer and dimerization can contribute to regulating editing activity and substrate specificity (143, 144). The minimum region required for the dimerization of Drosophila ADAR is the N-terminus including and the first dsRBD (144). Although the exact region required for dimerization of human ADAR is not known yet, the dsRBDs are likely to be involved as these domains often regulate protein dimerization (78, 145). Considering that ADAR1 can be modified by SUMO-1 in a region between the Z-DNA and first dsRBD and that this modification decreases editing activity, it is hypothesized that the SUMO modification sterically hinders dimerization, or interferes with substrate binding (146).

Another post-translation modification that affects ADAR1 function is ubiquitination. IFN signaling was shown to promote the Lys-48 mediated ubiquitination and degradation of p110 (147). Critically, this down-regulation was shown to be required for IFN signaling to execute efficient antiviral activity during VSV infection, suggesting that the cell needs to bypass the constant inhibitory effect of ADAR1 on IFN signaling in order to achieve an effective antiviral response. As p110 is largely absent from the cytoplasm, these data might suggest that newly transcribed unedited endogenous dsRNAs, exported from the nucleus into the cytoplasm, are involved in mounting a robust IFN response. It is tempting to speculate that these endogenous RNAs help stabilize RLR signaling complexes without requiring large amounts of harmful viral dsRNA.

Another mechanism by which ADAR1 could be modulated during viral infection is by regulating the availability of a free inositol pyrophosphate (IP6). IP6 may regulate ADAR1 as it is an essential cofactor for human ADAR2 and has been found buried within the enzyme core of the enzyme (148). In addition, most of the IP6 contact residues are conserved between human ADARs, and yeast ADAT1, a member of a family of related adenosine editing enzymes that act on transfer RNA (tRNA) also relied on IP6 for tRNA editing. Interestingly, a recent study that employed a human genome-wide RNA interference screen identified an essential role for inositol pyrophoshates in the type I IFN response as the activities of the inositol polyphosphate kinases, IPPK, PPIP5K1, and PPIP5K2 (which convert IP5 to IP6 and 1-IP7) were crucial for interferon induction and the control of Sendai and influenza A viruses (149). Whether this is linked to ADAR1 activity remains to be tested.

Altogether, these data indicate that ADAR1 activity can be controlled rapidly through protein-protein interactions, post-translational modifications, or the availability of cofactors. Downmodulation of ADAR1 activity may be expected early in infection to mount an effective IFN response, but later in infection an increase in ADAR1 activity may be necessary to prevent apoptosis in certain cell types. The discovery of novel mechanisms that regulate ADAR1 activity will be highly interesting for the treatment of viral infections, cancer, and autoimmune diseases.

## Concluding Remarks

**Figure 3:**
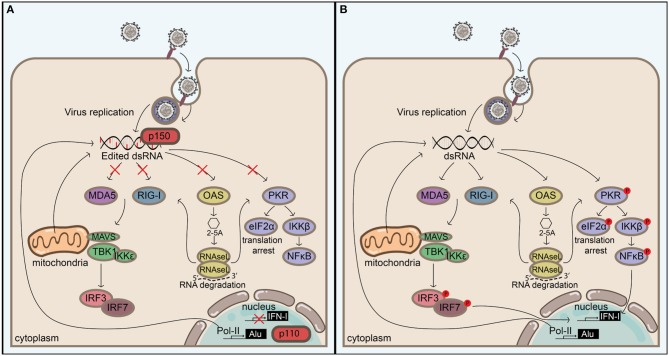
ADAR1 balances self-tolerance and immune activity by modulating canonical antiviral pathways induced by dsRNA. **(A)** Adenosine to inosine editing or binding of the cytoplasmic ADAR1 isoform p150 or the nuclear p110 to extended dsRNA duplexes prevents their detection by cytoplasmic antiviral signaling pathways, including RIG-I like receptor-, OAS/RNAseL-, and PKR pathways. These dsRNA duplexes can be of viral origin, but in the absence of ADAR1 **(B)** endogenous dsRNAs—which are likely to originate from inverted Alu repeats or other sources (e.g., mitochondrial dsRNAs)—can also serve as a substrate for antiviral signaling, leading to immune activation, and possibly autoimmunity. Blocking the activation of these pathways prevents IFN-I production, translation arrest, and apoptosis, but this must be tightly regulated in order to not create an environment that favors virus replication.

Extended, perfectly matching dsRNA duplexes are unusual in eukaryotic cells and are indicative of viral replication or the expression of endogenous mobile genetic elements. Sensory systems have evolved to respond to dsRNA. For example, the RLR signaling, PKR, and OAS-RNAse L systems all act as sensors and/or effectors in the response to dsRNA. Findings from studies over the past decade or so indicate that these pathways are all regulated by ADAR1 (Figure 3), which was originally discovered for its ability to unwind dsRNA structures. Although the mechanisms behind ADAR1's regulation of these pathways are currently under extensive investigation, it is likely that there are both editing-dependent and-independent mechanisms. The importance of ADAR1 in maintaining homeostasis is underlined by the severe autoimmune phenotype of AGS patients, which can be recapitulated through homozygous deletion of *Adar1* in mice. In agreement, recent advances confirm that the main function of ADAR1 seems to be to prevent self RNAs from triggering immune responses. The majority of these RNAs is likely to consist of Alu repeats, although the conceptual distinction between self and non-self seems to be complicated by such mobile genetic elements. Intriguingly, these repeats may be beneficial to their host by accelerating host evolution (150), and it seems that expression of ADAR1 allows us to live together with these selfish elements by blocking their intrinsic immunostimulatory molecular patterns. These molecular patterns are also present in viruses, which calls for the need for a tight regulation on A-to-I editing in order to not predispose the host for viral infections. In accordance, dysregulation of ADAR1 may play an important role in viral pathogenesis. In addition, these molecular patterns may be elevated in malignant cells, which may explain why these cells have higher editing activities. This could render malignant cells susceptible to cell death by ADAR1 inhibition and could pinpoint ADAR1 as a new target in immuno-oncology. Continued investigation of the function and regulation of ADAR1 will help identify mechanisms regulating the balance between immune activity and self-tolerance.

## Author Contributions

All authors listed have made a substantial, direct and intellectual contribution to the work, and approved it for publication.

### Conflict of Interest Statement

The authors declare that the research was conducted in the absence of any commercial or financial relationships that could be construed as a potential conflict of interest.
